# Detection of innate immune response modulating impurities (IIRMI) in therapeutic peptides and proteins: Impact of excipients

**DOI:** 10.3389/fimmu.2022.970499

**Published:** 2022-09-06

**Authors:** Seth G. Thacker, Cheng Her, Logan Kelley-Baker, Derek D C. Ireland, Mohanraj Manangeeswaran, Eric S. Pang, Daniela Verthelyi

**Affiliations:** ^1^ Laboratory of Immunology, Office of Biotechnology Products, Center for Drug Evaluation and Research, U.S. Food and Drug Administration, Silver Spring, MD, United States; ^2^ Division of Therapeutic Performance, Office of Research and Standards, Office of Generic Drugs, Center for Drug Evaluation and Research, U.S. Food and Drug Administration, Silver Spring, MD, United States

**Keywords:** immunogenicity, excipient, IIRMI, *In vitro* model, reporter cell lines, masking

## Abstract

Unintended immunogenicity can affect the safety and efficacy of therapeutic proteins and peptides, so accurate assessments of immunogenicity risk can aid in the selection, development, and regulation of biologics. Product- and process- related impurities can act as adjuvants that activate the local or systemic innate immune response increasing the likelihood of product immunogenicity. Thus, assessing whether products have innate immune response modulating impurities (IIRMI) is a key component of immunogenicity risk assessments. Identifying trace levels of individual IIRMI can be difficult and testing individually for all potential impurities is not feasible. Therefore, to mitigate the risk, cell-based assays that use human blood cells or monocyte-macrophage reporter cell lines are being developed to detect minute quantities of impurities capable of eliciting innate immune activation. As these are cell-based assays, there is concern that excipients could blunt the cell responses, masking the presence of immunogenic IIRMI. Here, we explore the impact of frequently used excipients (non-ionic detergents, sugars, amino acids, bulking agents) on the sensitivity of reporter cell lines (THP-1- and RAW-Blue cells) and fresh human blood cells to detect purified TLR agonists as model IIRMI. We show that while excipients do not modulate the innate immune response elicited by TLR agonists *in vivo*, they can impact on the sensitivity of cell-based IIRMI assays. Reduced sensitivity to detect LPS, FSL-1, and other model IIRMI was also evident when testing 3 different recombinant drug products, product A (a representative mAb), B (a representative growth factor), C (a representative peptide), and their corresponding formulations. These results indicate that product formulations need to be considered when developing and validating cell-based assays for assessing clinically relevant levels of IIRMI in therapeutic proteins. Optimization of reporter cells, culture conditions and drug product concentration appear to be critical to minimize the impact of excipients and attain sensitive and reproducible assays.

## Introduction

Proteins and peptides are key tools for the successful treatment of cancers, genetic defects, metabolic and infectious diseases. However, undesired immune responses elicited by these products can affect their safety or efficacy (1, 2). Indeed, product immunogenicity and subsequent changes in drug clearance profile, loss of efficacy, and even ensuing clinical deficiency syndromes have impeded the development and licensing of multiple therapeutics (3–5) and limit the clinical usefulness of others (6–8). Immunogenicity risk is determined by an incompletely understood set of patient- and product- related factors and discerning their individual impact is difficult; however understanding and controlling the known contributing factors can inform the risk for products, streamlining product development, licensure or approval, and clinical use (3, 4, 9).

One factor that can potentially be assessed and controlled is the presence of innate immune response modulating impurities (IIMRI) as these can induce local and systemic inflammation, contribute to hypersensitivity and development of adaptive responses to the product. These impurities can be found in biotechnology derived therapeutics despite careful downstream purification and can include trace levels of impurities such as: remnants of adventitious agents, host cell proteins (HCP), growth media components, host cell DNA (HCD) (10), leachates, and other contaminants (10–12). IIRMI can activate antigen presenting cells (APCs) such as macrophages and dendritic cells, which are embedded in subcutaneous tissues and line the endothelia. These cells are decorated with a broad array of pattern recognition receptors (PRR) and constantly sample their environment to identify danger or pathogen related signals. The broad array of innate immune receptors present on the cell surface, endosomes, and cytosol include Toll-like receptors, RIG-I and NOD-like receptors as well as C-type lectin and scavenger receptors (13–15). Their activation triggers an intracellular signaling cascade that leads to the activation of transcription factors such as NF-κB or AP1, which in turn increase the expression of proinflammatory genes leading to local inflammation and recruitment of other immune cells magnifying the immune response (16). Importantly, proteins that are up-taken together with PRR agonists are preferentially processed and presented in the context of MHC and costimulatory proteins, which are necessary to trigger antigen-specific T cell responses (17). Robust, prolonged, or repeated activation of the innate system can enable an immune response to nonimmunogenic or previously tolerized proteins or peptides (18, 19). Lastly, in a previous study using non-human primates we showed that the addition of very low levels of TLR ligands capable of inducing mild localized changes in the subcutaneous space where a therapeutic protein was administered correlated with increased anti-drug antibody (ADA) titers (20). Therefore, assessing whether there are impurities in therapeutic products capable of stimulating the innate immune system is critical to assessing their immunogenicity risk in support of product development and licensing (1, 2).

Implementation of assays to detect IIRMI is challenging as a broad range of potential product- and process- related impurities derived from raw materials or manufacturing processes can activate APCs even when present at trace levels. Further, there is evidence that multiple IIRMI can synergize with peptide or protein aggregates to foster immune responses (21–23). Given that generating highly sensitive assays to measure trace levels of unidentified individual impurities is very challenging, the methods currently used to screen products for the presence of IIRMI focus downstream by detecting activation markers on immune cells (10–12, 18, 22, 24–28). In our studies we have used the expression of mRNA for proinflammatory cytokines in human PBMC or the activation of NF-κB in monocytic cell lines that have multiple innate immune receptors, such as MM6, THP-1 or RAW cells, to detect IIRMI *in vitro* (29). However, a recent study suggested that product formulation could modify the results of IIRMI assay (30) raising concern that product excipients can alter the sensitivity of the IIMRI assays masking the presence of impurities.

Therapeutic peptides and proteins tend to be unstable and susceptible to aggregation, chemical or physical degradation, and are therefore carefully formulated to insure stability. Thus, a drug product consists of the active pharmaceutical ingredient (API) and its excipients. For aqueous-based injectable formulations, some of the most commonly used excipient types include buffering agents, stabilizers, tonicity adjustment agents, lyoprotectants and antioxidants as recently reviewed (31, 32). Sodium chloride, polysorbate 80, sucrose, and mannitol are the most common excipients after water, but most biologics have at least 4 components in their formulation (32). Moreover, the concentration of the different excipients can vary greatly between individual drug products. Importantly, excipients have been linked to injection site reactions and anaphylactoid responses (33–35). To examine whether excipients could mask the presence of impurities, we selected a group of frequently used excipients including surfactants (polysorbate 80, poloxamer 188), a stabilizer (albumin), sugars (sucrose, trehalose, mannitol), and amino acids (histidine, arginine), and explored their impact on cell-based assays to detect IIRMI. Further, we determined whether the assays could identify different purified IIRMI when spiked into 3 fully formulated commercial drug products. Our studies demonstrate product formulation is a critical parameter to consider when developing and validating cell-based assays for assessing potential IIRMIs in therapeutic proteins as demonstrated by: i) the excipients used in the formulation of proteins and peptides can interfere with the assessment of IIRMI in drug product; ii) the degree of masking depends on the nature of the impurities, as well as the cell platform and the excipients used; and iii) when using primary cells, the degree of masking varies by PBMC donor. This indicates that cell-based assays can be used to detect IIRMI but deriving actionable/interpretable data requires careful product-specific development and validation that ensures that the assay consistently detects the expected array and level of potential IIRMI.

## Materials and methods

### Reagents

LPS, Poly I:C, FSL-1, CLO75, CpG 1555 and zymosan were purchased from *In vivo*Gen (San Diego, CA). Sucrose, mannitol, trehalose, and L-arginine were obtained from Pfanstiehl (Waukegan, IL). All other chemicals were obtained from ThermoFisher Scientific (Waltham, MA) at USP grade. Commercial lots of Albucult (Albucult, Novozymes, Nottingham, UK) were used as a source of albumin. Excipients were dissolved in cell culture grade water (Lonza). The stock concentrations of excipients were made based on weight/weight and are expressed as a mass percentage or simply as percentage. The following drug products were used in our study: Product A (Bevacizumab, Genentech Inc. South San Francisco, CA), product B (Darbepoetin alpha, Amgen, Thousand Oaks, CA), and product C (glucagon, Eli Lilly, Indianapolis, IN). Drug formulation buffers were made based on details provided in the product insert and all components were of USP grade. All dilution percentages were calculated as weight/volume based on product insert information.

### Cell culture

#### PBMC isolation and stimulation

Deidentified fresh buffy coats were obtained from the NIH blood bank, (Bethesda, MD). PBMC were isolated by density-gradient centrifugation over Ficoll-Hypaque. PBMC were cultured at 37°C in RPMI medium + 10% FBS (heat inactivated), 1mM sodium pyruvate, 1X nonessential amino acids, 10mM HEPES, 100U penicillin/100ug streptomycin, and 2mM L-glutamine. PMBC were resuspended at a density of 4x10^6^ cells/mL. PBMCs (4x10^5^ cells/mL in 100μL) were added to 96 well plates followed by 50μL of a 40% (volume/volume) drug product or drug product formulation buffer with a final addition of 50uL of the test IIRMI resulting in cultures with 10% drug product. PBMCs were stimulated with trace levels of LPS (10pg/mL), FSL-1(100pg/mL), and Zymosan (1ng/mL) as model IIRMIs. Each condition was performed in triplicate. Following 24h of stimulation, PBMC were collected, lysed with TRIzol reagent (Invitrogen, Carlsbad, CA), and stored at -80°C before extracting RNA.

#### RAW-BLUE

Murine Raw 264.7 macrophages carrying a SEAP reporter construct inducible by NF-κB were purchased from *In vivo*Gen. Cells were grown in DMEM supplemented with 10% FCS, 2mM L-glutamine, 100μg/mL Normocin in the presence of selection antibiotic 200μg/mL Zeocin and passaged when 70% confluence was reached per manufacturer’s recommendation. Cells were scraped and resuspended in RAW-BLUE test media (DMEM, 10% heat inactivated FBS, 100μg/mL Normocin and 2mM L-glutamine) and plated at 100,000 per 96 well for testing.

#### THP-1-BLUE

THP-1 cells with a a SEAP reporter construct inducible by NF-κB were purchased from *In vivo*Gen. THP-1-reporter cells were maintained in RPMI-1640 media supplemented with 10% FBS, 10mM HEPES, β-mercaptoethanol, 2mM L-glutamine and Blasticidin selection antibiotic (*In vivo*Gen). Cells were washed and resuspend in test media (RPMI 10% heat inactivated FBS 2mM L-glutamine) and plated at 100,000 per 96 well for all tests.

#### qRT-PCR analysis

mRNA induction following stimulation of PBMCs was performed by qRT-PCR. Total RNA was prepared from cell lysate using TRIzol (Invitrogen, Carlsbad, CA) as per manufacturer instructions. RNA (500ng/mL) was reverse transcribed into cDNA using high-capacity cDNA Reverse Transcription Kit (Applied Biosystem, Foster City, CA) as per manufacturer recommendation. The qPCR reactions were carried out using Universal master mix and IL-8, IL-6, IL-1β, S100A8 and GAPDH primers (Applied Biosystem, Foster city, CA) in a Viia7 Real-time PCR system (Applied Biosystem, Carlsbad, CA). The relative expression levels of IL-8, IL-6, IL-1β, or S100A8 mRNA was calculated using the 2^-ΔΔCT^ method. Briefly, the calculation was performed using the following equations: ΔCT= CT(target gene) - CT(GAPDH), ΔΔCT = ΔCT(a target sample) − ΔCT(media, DP, or Formulation buffer alone), and Fold change was calculated using 2^^- ΔΔCT^.

#### Cell viability/metabolism

Cell viability/metabolism was measured using the CCK8 cell viability assay (Dojindo Molecular Technologies, Inc, Rockville, MD) per manufacture recommendations.

#### Testing of products on NF-κB reporter cells lines

RAW-Blue and THP-1-Blue cells were plated at 1x10^6^ cells/ml in flat bottom 96-well plates in a 100 µL volume. Excipients and TLR ligands were added for a final volume of 200 μL. After 24 h of stimulation, supernatants were collected and NF-κB activation was measured using the QUANTI-Blue detection medium according to manufacturer recommendations (*In vivo*Gen). Supernatants were read using Victor 3 plate reader (Perkin Elmer, Akron, OH) at 620nm.

#### Mice

C57BL/6 mice were purchased from the Jackson Laboratory. Mice were housed in the specific pathogen–free, Association for Assessment and Accreditation of Laboratory Animal Care International–accredited animal facility of the FDA’s Division of Veterinary Medicine (Silver Spring, Maryland, USA). This study was carried out in strict accordance with the recommendations in the Public Health Service Policy on Humane Care and Use of Laboratory Animals (Office of Laboratory Animal Welfare, 2015). Mice were shaved at least 24 hours prior to subcutaneous injection under anesthesia. On the day of inoculation mice were anesthetized and test articles were injected subcutaneously in a volume of 100 μL and the injection site was marked. After 6 hours the mice were euthanized and skin at the site of injection was collected. Skin biopsies were stored in Trizol until RNA was isolated using the manufactures recommendations.

#### Statistical analysis

The NF-κB induction in RAW- and THP-1-Blue cells was compared for each excipient tested using a two-way ANOVA followed by Dunnett’s test against the “unspiked control” with correction for multiple testing. The gene expression levels in PBMCs were calculated as the fold increase over the “unspiked control” or buffer using the 2^-ΔΔCt^ method. Differences in the IL-8 mRNA levels relative to the “unspiked control” or buffer were calculated using a one sample Wilcoxon test. All statistical comparisons were performed using GraphPad Prism 9.0. A p < 0.05 was considered significant. For multiple comparisons the stars in the graphs correspond to the post-test comparison with *p < 0.05 and **p < 0.01. For gene expression, the geometric mean and geometric standard deviation was calculated and presented in the text and graphs. Gene expression data are expressed throughout the text the values as geometric mean (geometric standard deviation) given that the distribution was not necessarily normal.

## Results

### Impact of excipients on the cell lines used to assess IIMRI

**Figure 1:**
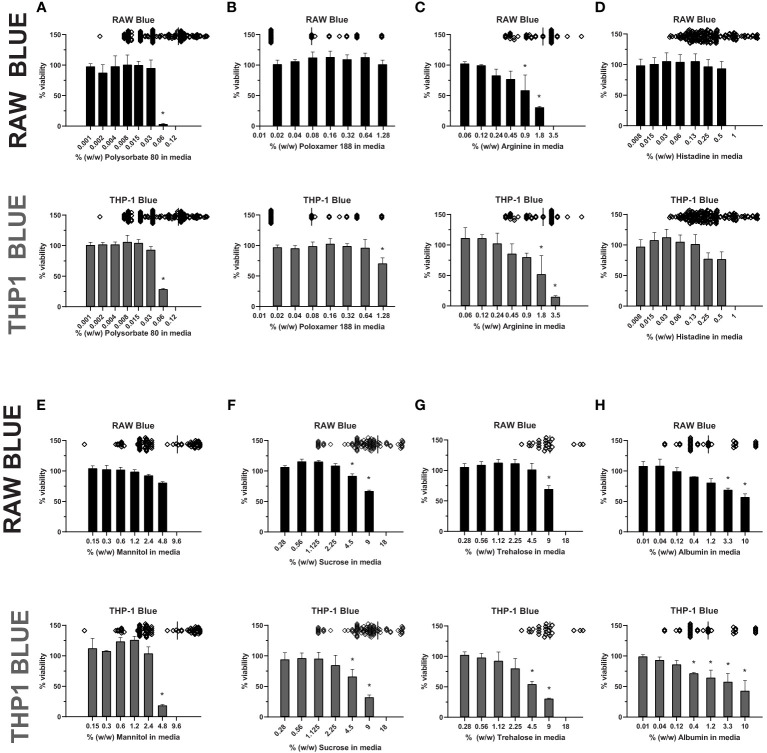
Excipient effect on cell viability. RAW-Blue and THP-1-Blue cells were incubated in triplicate with increasing concentrations of **(A)** Polysorbate 80, **(B)** Poloxamer 188, **(C)** Arginine, **(D)** Histidine, **(E)** Mannitol, **(F)** Sucrose, **(G)** Trehalose, or **(H)** Albumin for 24 hours. Top of each graph: Scatter plot depicting concentration of the excipient in licensed biological drugs regulated by the Office of Biotechnology Products at the FDA for reference. Cell metabolism/viability was assessed using the CCK8 assay. Results are shown relative to no-excipient control cells. The graph is representative of three independent experiments. Results are presented as the mean ± SD. RAW- and THP-1-Blue cells are solid black or grey, respectively. *p < 0.05.

Most therapeutic peptides and proteins are formulated using excipients to enhance manufacturability, stability, and delivery, however some excipients, like polysorbate 80, have been shown to modulate cell viability (33, 36, 37). To examine whether excipients impact the sensitivity of cell-based assays to detect IIRMI, we selected several commonly used excipients: polysorbate 80 and poloxamer 188 (surfactants), albumin (stabilizers), sucrose, trehalose and, mannitol (sugars), and histidine and arginine (amino acids) (31). Their impact on cell-based assays to detect IIRMI using Raw-Blue and THP-1-Blue cells was explored. These cells express multiple pattern recognition receptors and utilize a SEAP reporter to monitor NF-κB activation. The concentration of excipients was selected based on the range used in the formulation of currently licensed therapeutic proteins. To determine whether the different excipients could interfere with IIRMI assessments, we first established whether they would interfere with the viability of the reporter cells. Assessment of the impact of each excipient on cell viability/metabolism of the RAW- and THP-1-blue cell lines showed that most excipients reduced cell viability/metabolic activity at higher concentrations. As seen in **Figure 1**, concentrations of polysorbate 80 > 0.06% w/w (**Figure 1A**) significantly reduced cell viability while the cells tolerated higher levels of poloxamer 188 (**Figure 1B**) in both cell lines. For arginine, viability fell below 80% at concentrations higher than 0.9% w/w (**Figure 1C**), while histidine was tolerated at levels up to 0.5% w/w (**Figure 1D**). Mannitol had no significant impact at concentrations lower than 2.4% w/w in RAW and THP-1 cells (**Figure 1E**). Similarly, sucrose and trehalose impacted cell viability at concentrations > 2.25% w/w in THP-1 cells and 4.5% w/w in RAW cells (**Figures 1F–G**), while albumin reduced cell viability starting at 1.11% w/w and > 0.37% w/w for RAW and THP-1 respectively (**Figure 1H**). In both cell lines, altered metabolism/viability were seen at concentrations that are frequently used in licensed FDA products as seen by the scatter plots at the top of each graph (**Figure 1**). Indeed, only poloxamer 188 did not impact on RAW and THP-1 cells at levels used in licensed drug products. The decrease in metabolism/viability observed is not likely due to changes in the osmolarity of the culture media as the levels tested did not alter the osmolarity of the media and are more likely due to a direct effect of the excipient or an impurity in the excipient. Importantly, the quality of the excipient source has a major impact on cell viability/metabolism. Indeed, there were striking differences in the impact of excipients from different sources on cell metabolism/viability even though all reagents were listed as USP grade (**Supplementary Figure 1**). Since an impact on metabolic activity could confound the results, for all further studies we used concentrations of excipients that did not reduce metabolism/viability in the CCK8 assay by more than 20%.

### Impact of individual excipients on IIRMI assay sensitivity

**Figure 2:**
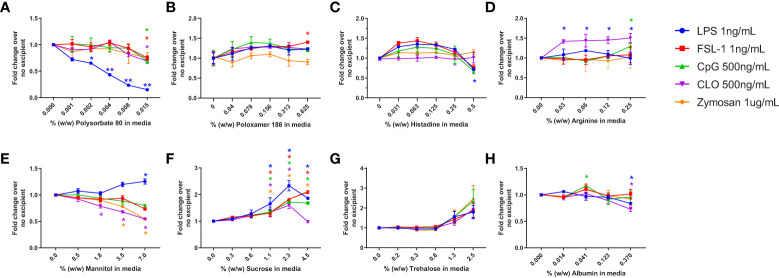
Impact of excipients on the sensitivity of RAW-Blue cells to detect IIRMI. RAW-Blue cells were stimulated with a single dose of different model IIRMI in the presence of increasing concentration of **(A)** Polysorbate 80, **(B)** Poloxamer 188, **(C)** Arginine, **(D)** Histidine, **(E)** Mannitol, **(F)** Sucrose, **(G)** Trehalose, or **(H)** Albumin for 24 hours. IIRMI activation of RAW-Blue cells was measured as NF-κB activation using Quanti-blue detection media. Graphs show the fold changes relative to the corresponding IIRMI stimulation in the absence of excipients. Cells were treated in triplicate and results are presented as the mean ± SD for 3 independent experiments. *p < 0.05 and **p < 0.01.

**Figure 3:**
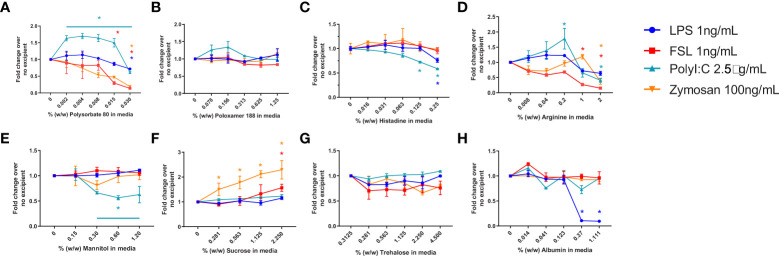
Impact of excipients on the sensitivity of THP-1-Blue cells to detect IIRMI. THP-1-Blue cells were stimulated with a single dose of model IIRMI in the presence of increasing concentration of **(A)** Polysorbate 80, **(B)** Poloxamer 188, **(C)** Arginine, **(D)** Histidine, **(E)** Mannitol, **(F)** Sucrose, **(G)** Trehalose, or **(H)** Albumin for 24 hours. IIRMI activation of THP-1-Blue cells was measured as NF-κB activation using Quanti-blue detection media. Graphs show fold changes relative to cells stimulated in the absence of excipients. Cells were treated in triplicate and results are presented as the mean ± SD of 3 independent experiments. *p < 0.05.

In a previous study we had established the limit of detection for individual TLR agonists when using RAW-and THP-1-Blue cells in our assay (29). To determine whether the presence of excipients modified the response to the purified TLR agonists we tested the 2 cell lines using increasing amounts of excipient and a fixed level of TLR agonist. The level of TLR agonists chosen to assess whether excipients modified the sensitivity of the assay were close to the lower range of detection but had previously induced a response consistently using these cells (29). Cells were stimulated with 1ng/mL of LPS (TLR4) or FSL-1 (TLR2), 2.5 µg/mL of poly I:C (TLR3), 500 ng/mL of CpG ODN 1555 (TLR9) or CL075 (TLR7), or 1μg/mL of Zymosan. As shown in **Figure 2**, RAW cells stimulated in the presence of increasing concentrations of polysorbate 80 showed a marked concentration-dependent reduction in NF-κB activation in response to LPS, while the activation by other TLR ligands was less impaired. For the other excipients, mannitol and albumin also tended to reduce the response to the TLR ligands at higher concentrations, while sucrose and trehalose increased the NF-κB response. Lastly, poloxamer 188 and arginine did not inhibit the response to the TLR ligands tested, but an increase in TLR7 response was observed. RAW cells were not stimulated with poly I:C as they do not express TLR3. THP-1 cells also showed a reduced NF-κB response to most TLR ligands in the presence of polysorbate 80, histidine, arginine, and trehalose, but the response was mostly unimpacted by poloxamer 188, mannitol, sucrose (except for zymosan) and albumin (**Figure 3**). Polysorbate 80, mannitol, histidine, and albumin modified the response to poly I:C, whereas polysorbate, arginine, and albumin reduced the response to LPS at higher concentrations (**Figure 3**). THP-1 cells do not express TLR7 or TLR9, so CLO75 and a CpG ODN were not used. Overall individual excipients can mask the presence of IIRMIs in both RAW-Blue cells and THP-1 cells, but the magnitude and effect depend on the excipient and cell line used.

### Effect of Polysorbate 80 on the response to IIRMI *in vivo*

**Figure 4:**
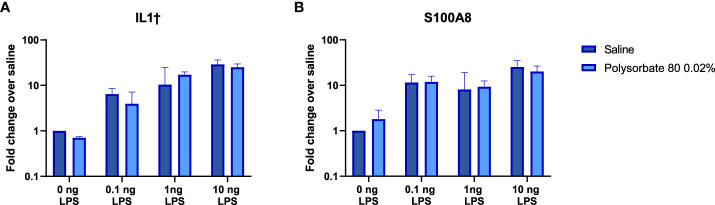
Polysorbate 80 does not mask detection of LPS *in vivo*. Mice were injected subcutaneously with increasing concentrations of LPS in the presence of saline (blue bars) or 0.02% w/w polysorbate 80 (light blue bars) (n=3/group). Results are shown as the fold change in IL-1β **(A)** and S100A8 **(B)** mRNA in local skin 6 hours post injection relative to mice that received the same volume of saline. Data is presented as the geometric mean and geometric standard deviation.

Given that the mechanism by which the excipients mask the presence of TLR agonists in RAW-Blue and THP-1 cells is not clear, we next determined whether the presence of the excipients would modify the innate immune response elicited by the TLR ligands when they were administered *in vivo*. Previous studies had shown that inoculation of low levels of TLR agonists induced local expression of proinflammatory genes (20). Polysorbate 80 was chosen to test *in vivo* as it demonstrated the most pronounced effect on masking LPS detection *in vitro*. C57Bl/6 mice were inoculated subcutaneously with 0.1ng-10ng of LPS alone or co-administered with polysorbate 80 (0.02% w/w) in 100 μL of saline. This concentration of polysorbate 80 was chosen as it is the median polysorbate 80 levels in licensed biologics using an internal FDA database. The local skin was harvested after six hours, and the RNA was isolated. As shown in **Figure 4**, the IL1β and S100A8 mRNA levels induced by LPS were similar in mice that received the LPS alone or together with polysorbate 80 at all concentrations tested (**Figure 4**). This suggests that polysorbate 80 does not modulate the activity of potential TLR4-triggering impurities *in vivo* and underscore the need to consider potential masking when quantifying the presence of IIRMI in drug products using cell-based assays.

### Impact of product formulation on IIRMI detection by RAW-Blue and THP-1-Blue cells

**Figure 5:**
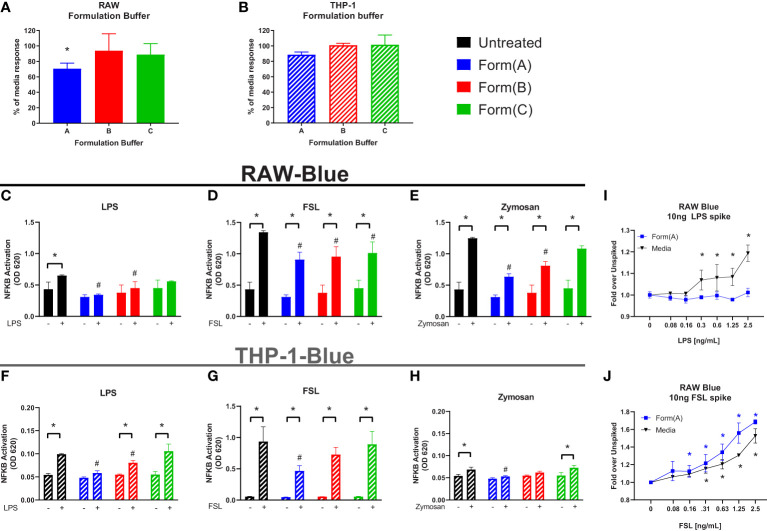
Impact of drug formulations on IIRMI detection by RAW-Blue and THP1 cells. RAW- and THP-1-Blue cells were stimulated with three model IIRMIs [(LPS (1ng), FSL (1ng), or Zymosan (1μg)] for 24 hours in the presence of drug product formulation (25% v/v). IIRMI activation of NF-κB in RAW- and THP-1-Blue cells was measured using Quanti-blue detection media. **(A–B)** Impact of 25% v/v drug formulation on baseline NF-κB activation in RAW- and THP-1-Blue cells. **(C–E)** RAW-Blue cells stimulated with IIRMIs for 24 hours in the presence of 25% v/v formulation. **(F–H)** THP-1-Blue cells stimulated with IIRMIs for 24 hours in the presence of 25% v/v formulation. **(I, J)** RAW-Blue cells were incubated for 24 hours with LPS or FSL in presence of 25% v/v formulation A or media alone. Results are shown relative to IIRMI stimulation in media alone (no formulation) treated cells. Results are representative of at least 3 independent experiments. Cells were treated in triplicate and results are presented as the mean ± SD. * or ^#^p <0.05 and **p < 0.01. * indicates statistical difference between spiked and unspiked cells in the corresponding formulation; # indicates statistical difference to the level of NF-κB activation by the corresponding stimuli in the absence of formulation (black bar, untreated).

Most formulations for biologics are complex and involve 4-5 excipients (32). Having shown that individual excipients can impact on the sensitivity of the IIRMI assays, we next examined whether masking occurs in the presence of formulations used by FDA licensed or approved products. To address this, we recreated the formulation for 3 FDA-licensed therapeutic products that lack overt immunomodulatory effects: product A (a Mab), product B (a growth factor), and product C (a peptide) that ranged in size from 149kDa to 3.4kDa using USP grade chemicals (**Table 1**). The formulation for product A contains trehalose as well as polysorbate 20, while that of product B contains polysorbate 80. The formulation for product C is simpler, containing only lactose but with a highly acidic pH. Replacement of 25% of the culture media volume with formulation buffer did not reduce cell metabolism/viability below 95% (**Supplementary Figures 2A, B**), however the SEAP level in unstimulated cells in culture for 24 h was reduced in the presence of product A formulation (**Figures 5A, B**). When the cells were stimulated with LPS (100pg/ml), FSL-1 (100pg/ml), and Zymosan (1μg/ml) in the culture media with 25% formulation buffer for 24 h, the increase in NF-κB activation was significantly lower than that observed in cells that had not received formulation buffer particularly for the cells stimulated in the presence of product A or B formulation. Importantly, the product A formulation masked the presence of LPS when using both cell lines and both product A and B masked the presence of Zymosan in THP-1-but not RAW-Blue cells. Of note, addition of product A formulation masked the presence of up to 2.5 ng/ml of LPS but sensitivity to FSL-1 was not impacted (**Figures 5I, J**). This suggests that masking is not uniform and can impact different receptors or innate immune pathways differently.

**Table 1:** Drug formulation composition.

Drug Product	Formulation component (concentration w/w)
Product A (pH 6.2)	Trehalose (6%) Na Phosphate, monobasic (0.58%) Na Phosphate, dibasic (0.012%) Polysorbate 20 (0.04%)
Product B (pH6.2)	NaCl (0.818%) Na Phosphate, monobasic (0.212%) Na Phosphate, dibasic (0.066%) Polysorbate 80 (0.005%)
Product C (pH 2.5-3.5)	Lactose (10.7%)

### Detection of IIRMI in formulated therapeutic proteins and peptides by PBMCs

**Figure 6:**
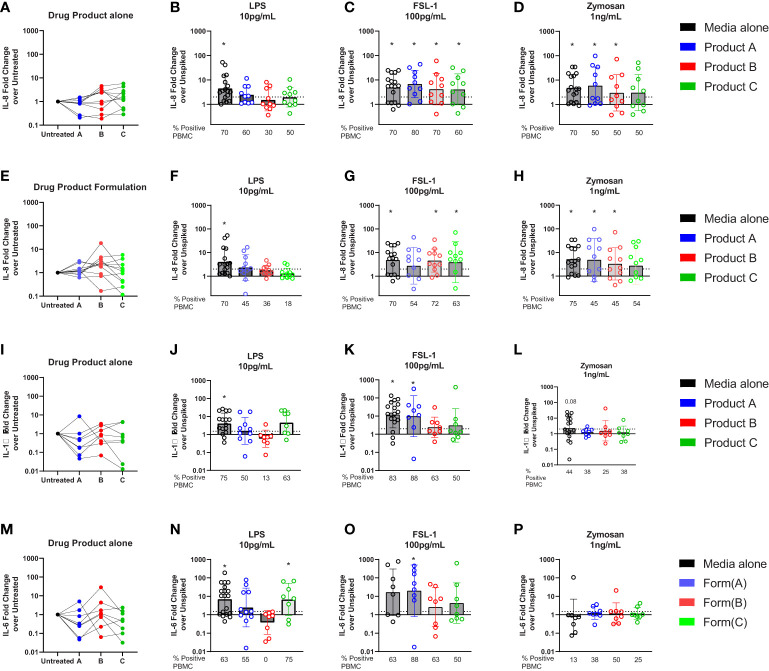
Impact of drug formulations on PBMCs *in vitro* detection of IIRMI. PBMCs were stimulated for 24h with LPS (10pg), FSL (100pg), or Zymosan (1ng) in media alone, drug product or the corresponding formulation (10% v/v) for 3 licensed or approved products. Fold changes in IL-8 gene expression are relative to the unspiked cells. Fold increase in mRNA for IL-8 **(A–E)**, IL-6 **(I)**, and IL-1β **(M)** expression by PBMC treated with drug product **(B-D, J-P)** or drug formulation **(F-H)** (10% v/v) unspiked or spiked with TLR agonists. N=8-18 PBMC from healthy donors per condition. Donors were considered positive if the level of mRNA expression was ≥ 2-fold increase over the unspiked control; the percentage of positive PBMC in each condition is presented under each graph. * indicated p < 0.05 and is a comparison between the expression in cells stimulated with a model IIRMI in media, drug products, or drug formulation and the corresponding unspiked control.

Previous studies have shown that human PBMCs are activated by lower levels of TLR ligands than monocytic cell lines and are being used to monitor IIRMI (18, 29, 38). Therefore, we examined whether drug product or the corresponding formulations for product A, B, or C impacted detection of IIRMI when using fresh PBMCs from 10 donors. The concentration of the drug product added to the wells was 10% (v/v) to minimize shifts in osmolarity, pH, or dilution of the media; this was reduced from 25% volume replacement of media that was used when assessing IIRMI with the THP-1- and RAW-Blue cell lines based on reduced viability of PBMCs in the presence of drug product. As shown in **Supplementary Figure 2**, addition of 10% v/v drug product or product formulation did not impact cell viability as assessed by CCK8. The expression of mRNA for IL-8, IL-6 or IL-1β were used as a measurement of innate immune activation as previous studies had shown that they were broadly induced by multiple IIRMI *in vitro* and *in vivo* and results are expressed as fold change over the corresponding unspiked control for each donor (20, 29). Changes in mRNA expression >2 were considered positive. Addition of these therapeutics to PBMCs in culture as expected did not modify the expression of IL-8, IL-6 or IL-1β mRNA, although there was increased variability in mRNA levels, particularly with the cells incubated in the presence of product C (**Figures 6A, E, I**).

To determine whether the therapeutics would mask recognition of impurities triggering TLRs, cells were stimulated with low levels of LPS (10pg/ml), FSL-1 (100pg/ml), and Zymosan (1ng/ml) alone, or in the presence of drug product and induction of the Il8, Il6 and Il1β mRNA was assessed. The concentration of TLR ligands chosen was close to the known limit of detection for the assay (29) and in the absence of drug product or drug formulation the selected concentrations of TLR ligands induced a modest but consistent induction of IL8 mRNA (LPS (4.5(3.3)), FSL(4.9(3.4)), and Zymosan(4.8(3.6)), **Figures 6B–D**). However, as shown in **Figure 5B** for RAW and THP-1 cells, in PBMCs there was an overall reduction in the magnitude of the response and the percentage of donors that had a greater than 2-fold increase in IL8 (**Figure 6B**), particularly for products spiked with LPS. The decrease was most striking for the cells exposed to product B, which showed a mean fold change of 1.5(2.8) and where only 30% of donors responded to 10pg/mL LPS stimulation (**Figure 6B**). Products A and C also reduced the number of responders to 10pg/mL LPS from 70% of samples in the absence of drugs, to 60 and 50%, respectively (**Figure 6B**). However, stimulation of PBMCs with 100pg/mL FSL-1 was not impacted by the presence of product A, B or C (**Figure 6C**). In PBMCs stimulated with 1ng/mL of Zymosan, the number of PBMCs samples with >2-fold change in IL8 mRNA levels relative to the corresponding unspiked controls was reduced from 70 to 50%, although the mean fold change in IL-8 expression was different from the unspiked cells for product A and B (**Figure 6D**). Together this indicates that the presence of 10% drug product in the culture masked the presence of LPS, but not FSL-1. The response to zymosan was preserved except in the presence of product C formulation (**Figure 6D**).

To determine whether masking was associated with the presence of the corresponding API, or solely the result of the drug formulation, we next repeated the study using product A, B, and C drug formulation (no API). As shown in **Figures 6F–H**, the results were comparable to those observed when using the drug product indicating that the masking results from the effect of the excipients in the product and not the API.

Similar trends were observed when measuring IL-1β or IL-6 as a readout for the response to LPS and FSL-1 (**Figures 6J, K, N–O**) although the increase in mRNA expression was muted in cells stimulated with Zymosan (**Figures 6H, L**). The impact of drug formulation on IL-1β or IL-6 is shows similar trends as the drug product (**Supplementary Figure 3**). Together these results demonstrate the importance of choosing the right readouts when monitoring for the presence of potential IIRMI to ensure adequate sensitivity to potential IIRMI.

**Figure 7:**
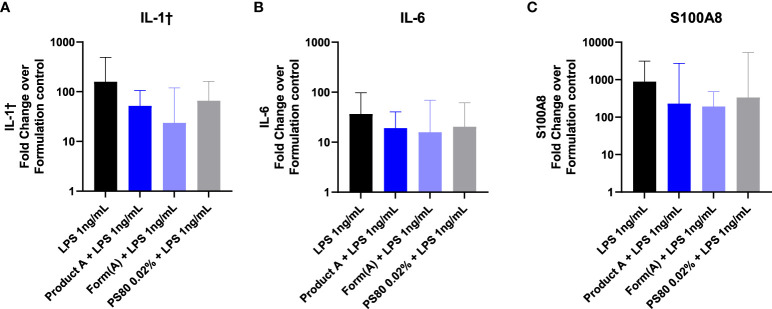
Product A does not mask detection of LPS *in vivo*. Mice were injected subcutaneously with 1ng of LPS with saline, product A, the formulation A, or polysorbate 80. The induction of IL-1β **(A)**, IL-6 **(B)**, and S100A8 **(C)** at the site of injection was measured in skin 6 hours post injection. Results are shown as the fold increase over saline alone. Data is presented as the geometric mean and geometric SD. n = 4-5.

Lastly, we examined whether the drug product or its formation modulate the effect of TLR inoculation into the subcutaneous space. Mice were inoculated with 100 μL of LPS (1ng) in saline, in product A, in product A’s formulation, or in polysorbate 80 (0.02%). Inoculation of LPS induced a robust upregulation of IL1β, IL6, and S100A8 at the inoculation site that was detectable 6 hours after challenge (**Figure 7**). The induction of IL1β, IL6, or S100A8 in mice that received LPS was evident regardless of whether the LPS was inoculated together with product A, its complete formulation, or polysorbate 80 was not significantly different than LPS alone. These results confirm that despite the masking effect of formulation *in vitro*, it does not modulate the impact of impurities *in vivo*.

## Discussion

IIRMI can derive from any step in the manufacturing process, from raw materials to container closure systems, and encompass not only remnants of adventitious agents, host cells, or tissue components but also residual compounds used in peptide or oligonucleotide synthesis such as dithiothreitol, or leachates or elemental impurities from container closure capable of activating antigen presenting cells (39, 40). Product aggregates, proteinaceous or extraneous particulates can also activate the cells of the innate immune system (41). Most importantly, many of these impurities can act in concert to modulate the activity of the local immune cells and foster local or systemic responses, although the precise magnitude or persistence of the response needed to elicit an adaptive immune response is still unclear (22, 42–45). Lastly, some commonly used excipients such as such as polysorbate 80 (46) (22, 42–45), mannitol (47), or sucrose (41) can potentially modify the activation of antigen presenting cells directly or through degradation products (48). Compendial tests exist to control the levels of a few impurities such as LPS (LAL assay), nucleic acids, PS degradation, and b glycans, but sensitive tests that can detect the presence of a broader array of IIRMI or gage whether different impurities might synergize to activate the innate immune system are lacking.

Cell-based assays that measure innate immune activation can be utilized to determine whether therapeutic products contain IIRMI. Assays using monocyte, macrophages, and other immune cells have the capacity to detect many potential IIRMI due their expression of PRRs such as TLRs, MAVS, STING, and NOD-like receptors and convey the additive effect of any IIRMI(s) in the culture (29, 38, 49). Multiple parameters can impact the choice of platform: Assays relying on whole blood or PBMCs tend to be more sensitive and capture a broader range of IIRMI, however they are harder to validate, difficult to store and retrieve and the frequency and responsiveness of cells of interest, including dendritic cells, mast cells and basophils may vary widely between samples. Assays based on monocytic cell lines are easier to implement and, as shown above, may tolerate a higher concentration of drug product without altering viability; however, the range of potential impurities they capture is more limited including a poor response to protein aggregates as compared to primary cells. The current studies suggest that the impact of drug formulation on assay performance can also impact on the choice of the assay platform and the sensitivity of the assay.

The formulation of biologics can be complex and different therapeutic products contain a wide range of excipients to stabilize and protect the API (31). Most commonly used excipients have a long history of safety, but some, including mannitol (50–52), polysorbates and its degradants have been associated with changes in product immunogenicity, injection site reactions, and hypersensitivity responses (33, 46, 53–55). Even sugars, most often thought to be benign, can be toxic if present at high enough concentrations or containing impurities (41, 56). In addition to their effects described above, our data shows that these excipients can impact the activation of an NF-κB response in monocyte/macrophages and impact on cell viability when present at concentrations often used in the formulation of FDA licensed or approved products. Specifically, some excipients such as polysorbate 80 can mask the presence of TLR ligands, particularly LPS, even when present at concentrations that do not change cell viability (**Figures 2**, **3**). This is consistent with a previous study assessing the impact of polysorbate 80 on the viability of HaCaT cells showed even less tolerance to the excipient (37). Conversely, products formulated with polysorbate 80 commonly use cell-based potency assays; however, these differences in the response to polysorbate 80 may be rooted in the type of cell line, the culture conditions, and concentration of drug product used. It should be noted that RAW-, THP-1-Blue and PBMCs did not show reduced viability when cultured in the presence of 10% v/v formulation buffer for any of the drugs tested (**Supplementary Figure 2**). A limitation of our current study is that we have not extensively tested multiple lots of each excipient or drug product to determine if the effects we are observing will vary from lot to lot. Our results suggest the need to test the suitability of the cells used to test IIRMI for each drug product, as failure to account for potential effects of excipients could lead to the under estimation of the presence of IIRMIs in therapeutic products.

Beyond cell viability, different excipients can impact on the response to IIRMI. Our current study shows that in monocyte/macrophage cell culture individual excipients such as polysorbate 80 (**Figures 2**, **3**) can inhibit the response to some TLR ligands, particularly LPS. This occurred at levels that were not overtly toxic to the cells. It is not clear at this point how the excipients modify the sensitivity of the IIRMI assay, but the effect appeared to be dependent on the concentration of the excipients. Possible mechanisms include physical masking, modifying the ligand as described for the effect of surfactants on LPS (57), interfering with the ability of receptors to recognize the ligands, inducing a downregulation of the expression of the receptors, or altering the metabolic state of the cells to dampen their response to the stimuli (58–61). Interestingly, despite these possibilities, the majority of the excipients tested did not impact the sensitivity of the IIRMI assays and, in some cases such as sucrose, magnified the response. LPS masking or low endotoxin recovery has been previously described both for cell-based (61) and cell-independent assays (57, 62), and multiple studies suggest that it depends heavily on the testing process (57, 61, 63). In agreement with this, our results suggest that IIRMI masking is an artifact of *in vitro* testing and does not translate into a muted response to the impurities *in vivo*. This is consistent with previous observations that salts (64), metal ions (65), carrier proteins, and other ECM components (66) may interfere with micelle formation and masking particularly for LPS and/or facilitate access to the pattern recognition receptors *in vivo*. In addition, excipients may by quickly diluted or taken up by cells in the subcutaneous space to levels that no longer interfere with the stimulation. On the other hand, if the masking is due to a direct effect of the excipients on the responsiveness of the immune cells, the number of potential responding cells *in vitro* is limited. Although not thoroughly investigated in these studies, it is likely that the impact of product formulation on IIRMI detection may not be directly inferred from the assessment of the individual formulation components, suggesting that the sensitivity of the IIRMI assay may need to be confirmed or even optimized for each product to reduce the impact of the product formulation on the chosen assay platform.

IIRMI assays are currently being used early in development as part of a pre-clinical immunogenicity risk assessment strategy and can be submitted to the regulatory agencies as part of the integrated summary of immunogenicity in an IND or EU Investigational Medicinal Product Dossier (IMPD) (67). In addition, IIRMI assays can be used in generic drug product development to support the potential immunogenicity risk of the generic product is not greater than the brand-name drug product it is referencing (68). Given that the aim of these assays is to understand whether the products administered to the patient contain impurities capable of stimulating immune cells at the site of administration, testing is often conducted using formulated drug product in its pertinent container closure. In these cases, it seems critical to understand the potential impact of the product formulation on assay performance through careful product-specific development and validation that ensures that the assay consistently detect the expected array and level of potential IIRMI in order to interpret the assay results and obtain meaningful data.

## Data availability statement

The original contributions presented in the study are included in the article/**Supplementary Materials**. Further inquiries can be directed to the corresponding author.

## Ethics statement

The studies involving human participants were reviewed and approved by FDA Institutional Review Board (IRB). The patients/participants provided their written informed consent to participate in this study. The animal study was reviewed and approved by Public Health Service Policy on Humane Care and Use of Laboratory Animals.

## Author contributions

ST, CH, MM, DI, EP, and DV contributed to conception and design of the study. LB performed the statistical analysis. ST and DV wrote the manuscript. All authors contributed to manuscript revision, read, and approved the submitted version.

## Funding

This study was supported in part by the Oak Ridge Institute for Science and Education through an interagency agreement between the US Department of Energy and the US Food and Drug Administration, FDA’s Center Of Excellence in Immunology (I-COE).

## Conflict of interest

The authors declare that the research was conducted in the absence of any commercial or financial relationships that could be construed as a potential conflict of interest.

## Publisher’s note

All claims expressed in this article are solely those of the authors and do not necessarily represent those of their affiliated organizations, or those of the publisher, the editors and the reviewers. Any product that may be evaluated in this article, or claim that may be made by its manufacturer, is not guaranteed or endorsed by the publisher.

## Supplementary material

Supplementary Figure 1 Excipient source has an impact on cell viability. RAW-Blue cells were incubated with increasing concentrations of excipients from 2 different suppliers for 24 hours. Cell metabolism/viability was assessed using the CCK8 assay. Results are shown relative to media alone treated cells. Results are presented as the mean ± SD. * p <0.05 and ** p < 0.01.

Supplementary Figure 2 Drug product and Drug product formulations effects on cell viability. RAW-Blue **(A)**, THP-1-Blue **(B)**, and PBMCs **(C, D)** were incubated in triplicate with the addition of 25% volume replacement of drug product or formulation alone for 24 hours. Cell lines were treated with 25% formulation volume replacement of media and PBMCs were incubated with 10% product or formulation volume replacement of media. Cell metabolism/viability was assessed using the CCK8 assay. Results are shown relative to the same cells cultured in media alone. Results are presented as the mean ± SD. RAW-, THP-1-Blue, and PBMC are solid, diagonal lines, or with vertical lines, respectively. ** p < 0.01.

Supplementary Figure 3 Drug formulation components can affect PMBCs ability to respond to IIMRIs PBMCs were stimulated for 24h with LPS (10pg), FSL (100pg), or Zymosan (1ng) in media alone or the corresponding formulation (10% v/v) for 3 licensed or approved products. Fold changes in IL-6 or IL-1β gene expression are relative to the unspiked cells. **(A–E)** show changes in IL-6 and IL-1β expression in PBMC in the presence or absence of drug product or drug formulation (10% v/v; unspiked with TLR agonists), respectively. **(B–D)** show changes in IL-6 mRNA expression and F-H show changes in IL-1β in PBMC (spiked) where 10% of media was replaced by drug formulation. N = 8-18 PBMC from healthy donors per condition. Donors were considered positive if the level of IL-8 was ≥ 2-fold increase over the unspiked control; the percentage of positive PBMC in each condition is presented under each graph. * indicated p < 0.05 and is a comparison between the stimulation of cells with a model IIRMI in media or drug formulation to the corresponding unspiked control.

## References

[1] FDA . Guidance for industry: Immunogenicity assessment for therapeutic protein products. In: Administration CfDEaRCCfBEaRCFaD. Silver Spring, Maryland: Food and Drug Administration. (2014).

[2] EMA . Guideline on immunogenicity assessment of therapeutic proteins In: Party BMPW, editor. 01/06/2017 ed. 30 Churchhill Place Conary Wharf London UK: European Medicines Agency (2017).

[3] Mahlangu JN Weldingh KN Lentz SR Kaicker S Karim FA Matsushita T . Changes in the amino acid sequence of the recombinant human factor VIIa analog, vatreptacog alfa, are associated with clinical immunogenicity. J Thromb haemost.: JTH (2015) 13(11):1989–98. doi: 10.1111/jth.13141 26362483

[4] Casadevall N Nataf J Viron B Kolta A Kiladjian J-J Martin-Dupont P . Pure red-cell aplasia and antierythropoietin antibodies in patients treated with recombinant erythropoietin. N Engl J Med (2002) 346(7):469–75. doi: 10.1056/NEJMoa011931 11844847

[5] Ridker PM Tardif JC Amarenco P Duggan W Glynn RJ Jukema JW . Lipid-reduction variability and antidrug-antibody formation with bococizumab. N Engl J Med (2017) 376(16):1517–26. doi: 10.1056/NEJMoa1614062 28304227

[6] Kalden JR Schulze-Koops H . Immunogenicity and loss of response to TNF inhibitors: implications for rheumatoid arthritis treatment. Nat Rev Rheumatol (2017) 13(12):707–18. doi: 10.1038/nrrheum.2017.187 29158574

[7] Petkau AJ White RA Ebers GC Reder AT Sibley WA Lublin FD . Longitudinal analyses of the effects of neutralizing antibodies on interferon beta-1b in relapsing-remitting multiple sclerosis. Mult Scler (2004) 10(2):126–38. doi: 10.1191/1352458504ms1004oa 15124756

[8] Scott DW Pratt KP . Factor VIII: Perspectives on immunogenicity and tolerogenic strategies. Front Immunol (2020) 10. doi: 10.3389/fimmu.2019.03078 PMC6978909 32010137

[9] Chung CH Mirakhur B Chan E Le QT Berlin J Morse M . Cetuximab-induced anaphylaxis and IgE specific for galactose-alpha-1,3-galactose. N Engl J Med (2008) 358(11):1109–17. doi: 10.1056/NEJMoa074943 PMC2361129 18337601

[10] de Zafra CLZ Quarmby V Francissen K Vanderlaan M Zhu-Shimoni J . Host cell proteins in biotechnology-derived products: A risk assessment framework. Biotechnol Bioeng (2015) 112(11):2284–91. doi: 10.1002/bit.25647 26010760

[11] Jiang Y Nashed-Samuel Y Li C Liu W Pollastrini J Mallard D . Tungsten-induced protein aggregation: solution behavior. J Pharm Sci (2009) 98(12):4695–710. doi: 10.1002/jps.21778 19645002

[12] Seidl A Hainzl O Richter M Fischer R Böhm S Deutel B . Tungsten-induced denaturation and aggregation of epoetin Alfa during primary packaging as a cause of immunogenicity. Pharm Res (2012) 29(6):1454–67. doi: 10.1007/s11095-011-0621-4 PMC3349029 22094831

[13] West AP Koblansky AA Ghosh S . Recognition and signaling by toll-like receptors. Annu Rev Cell Dev Biol (2006) 22(1):409–37. doi: 10.1146/annurev.cellbio.21.122303.115827 16822173

[14] Chen G Shaw MH Kim Y-G Nuñez G . NOD-like receptors: Role in innate immunity and inflammatory disease. Annu Rev Pathol.: Mech Dis (2009) 4(1):365–98. doi: 10.1146/annurev.pathol.4.110807.092239 18928408

[15] Creagh EM O’Neill LAJ . TLRs, NLRs and RLRs: a trinity of pathogen sensors that co-operate in innate immunity. Trends Immunol (2006) 27(8):352–7. doi: 10.1016/j.it.2006.06.003 16807108

[16] Hammer GE Ma A . Molecular control of steady-state dendritic cell maturation and immune homeostasis. Annu Rev Immunol (2013) 31:743–91. doi: 10.1146/annurev-immunol-020711-074929 PMC4091962 23330953

[17] Blander JM . Regulation of the cell biology of antigen cross-presentation. Annu Rev Immunol (2018) 36:717–53. doi: 10.1146/annurev-immunol-041015-055523 PMC6430635 29490164

[18] Wen Y Jawa V . The impact of product and process related critical quality attributes on immunogenicity and adverse immunological effects of biotherapeutics. J Pharm Sci (2021) 110(3):1025–41. doi: 10.1016/j.xphs.2020.12.003 33316242

[19] Verthelyi D Wang V . Trace levels of innate immune response modulating impurities (IIRMIs) synergize to break tolerance to therapeutic proteins. PloS One (2010) 5(12):e15252. doi: 10.1371/journal.pone.0015252 21203556 PMC3008684

[20] Haile LA Puig M Polumuri SK Ascher J Verthelyi D . *InVivo* effect of innate immune response modulating impurities on the skin milieu using a macaque model: Impact on product immunogenicity. J Pharm Sci (2017) 106(3):751–60. doi: 10.1016/j.xphs.2016.11.001 27923493

[21] Mäkelä SM Strengell M Pietilä TE Osterlund P Julkunen I . Multiple signaling pathways contribute to synergistic TLR ligand-dependent cytokine gene expression in human monocyte-derived macrophages and dendritic cells. J Leukoc Biol (2009) 85(4):664–72. doi: 10.1189/jlb.0808503 19164128

[22] Polumuri SK Haile LA Ireland DDC Verthelyi D . Aggregates of IVIG or avastin, but not HSA, modify the response to model innate immune response modulating impurities. Sci Rep (2018) 8(1):11477. doi: 10.1038/s41598-018-29850-4 30065306 PMC6068171

[23] van Haren SD Dowling DJ Foppen W Christensen D Andersen P Reed SG . Age-specific adjuvant synergy: Dual TLR7/8 and mincle activation of human newborn dendritic cells enables Th1 polarization. J Immunol (Baltimore Md: 1950) (2016) 197(11):4413–24. doi: 10.4049/jimmunol.1600282 PMC7386828 27793997

[24] Rosenberg AS . Effects of protein aggregates: An immunologic perspective. AAPS J (2006) 8(3):E501–E7. doi: 10.1208/aapsj080359 PMC2761057 17025268

[25] Rosenberg AS . Immunogenicity of biologicals therapeuticals: a heirarchy of concerns. Dev Biol (Basel). (2003) 112:15–21. 12762500

[26] Rosenberg AS Verthelyi D Cherney BW . Managing uncertainty: A perspective on risk pertaining to product quality attributes as they bear on immunogenicity of therapeutic proteins. J Pharm Sci (2012) 101(10):3560–7. doi: 10.1002/jps.23244 22736548

[27] Cohen S Myneni S Batt A Guerrero J Brumm J Chung S . Immunogenicity risk assessment for biotherapeutics through *in vitro* detection of CD134 and CD137 on T helper cells. mAbs (2021) 13(1):1898831. doi: 10.1080/19420862.2021.1898831 33729092 PMC7993230

[28] Tokuda JM Xie J Jawa V Hawkins JM Ferbas J Joh NH . Use of *In vitro* human skin models to assess potential immune activation in response to biotherapeutic attributes and process-related impurities. J Pharm Sci (2022) 111(4):1012–23. doi: 10.1016/j.xphs.2022.02.001 35139332

[29] Haile LA Puig M Kelley-Baker L Verthelyi D . Detection of innate immune response modulating impurities in therapeutic proteins. PloS One (2015) 10(4). doi: 10.1371/journal.pone.0125078 PMC4406594 25901912

[30] Holley CK Cedrone E Donohue D Neun BW Verthelyi D Pang ES . An in vitro assessment of immunostimulatory responses to ten model innate immune response modulating impurities (IIRMIs) and peptide drug product, teriparatide. Molecules (2021) 26(24):7461. doi: 10.3390/molecules26247461 PMC8707785 34946542

[31] Rao VA Kim JJ Patel DS Rains K Estoll CR . A comprehensive scientific survey of excipients used in currently marketed, therapeutic biological drug products. Pharm Res (2020) 37(10):200. doi: 10.1007/s11095-020-02919-4 32968854 PMC9010397

[32] Ionova Y Wilson L . Biologic excipients: Importance of clinical awareness of inactive ingredients. PloS One (2020) 15(6):e0235076. doi: 10.1371/journal.pone.0235076 32584876 PMC7316246

[33] Perino E Freymond N Devouassoux G Nicolas JF Berard F . Xolair-induced recurrent anaphylaxis through sensitization to the excipient polysorbate. Ann Allergy Asthma Immunol (2018) 120(6):664–6. doi: 10.1016/j.anai.2018.02.018 29481891

[34] Carbonell A Escudero AI Miralles JC González A Navarro C Cardona P . Anaphylaxis due to poloxamer 238. J Investig Allergol Clin Immunol (2018) 28(6):419–20. doi: 10.18176/jiaci.0298 30530388

[35] Pitlick MM Park MA Gonzalez-Estrada A Chiarella SE . Biphasic anaphylaxis after first dose of messenger RNA coronavirus disease 2019 vaccine with positive polysorbate 80 skin testing result. Ann Allergy Asthma Immunol (2021) 127(4):498–9. doi: 10.1016/j.anai.2021.07.020 PMC8325373 34343674

[36] Hirama S Tatsuishi T Iwase K Nakao H Umebayashi C Nishizaki Y . Flow-cytometric analysis on adverse effects of polysorbate 80 in rat thymocytes. Toxicology (2004) 199(2-3):137–43. doi: 10.1016/j.tox.2004.02.017 15147788

[37] Brosin A Wolf V Mattheus A Heise H . Use of XTT-assay to assess the cytotoxicity of different surfactants and metal salts in human keratinocytes (HaCaT). a feasible method for *in vitro* testing of skin irritants. Acta Derm Venereol (1997) 77(1):26–8. doi: 10.2340/0001555577026028 9059672

[38] Mufarrege EF Haile LA Etcheverrigaray M Verthelyi DI . Multiplexed gene expression as a characterization of bioactivity for interferon beta (IFN-beta) biosimilar candidates: Impact of innate immune response modulating impurities (IIRMIs). AAPS J (2019) 21(2):26. doi: 10.1208/s12248-019-0300-7 30737590

[39] Jenke DR Stults CLM Paskiet DM Ball DJ Nagao LM . Materials in manufacturing and packaging systems as sources of elemental impurities in packaged drug products: A literature review. PDA J Pharm Sci Technol (2015) 69(1):1–48. doi: 10.5731/pdajpst.2015.01005 25691713

[40] Qiu D Tan WC . Dithiothreitol has a dose-response effect on cell surface antigen expression. J Allergy Clin Immunol (1999) 103(5):873–6. doi: 10.1016/S0091-6749(99)70432-X 10329822

[41] Grabarek A Nabhan M Turbica I Hawe A Pallardy M Jiskoot W . Immunological evaluation in vitro of nanoparticulate impurities isolated from pharmaceutical-grade sucrose. J Pharm Sci (2021) 110(2):952–8. doi: 10.1016/j.xphs.2020.11.011 33220239

[42] Moussa EM Panchal JP Moorthy BS Blum JS Joubert MK Narhi LO . Immunogenicity of therapeutic protein aggregates. J Pharm Sci (2016) 105(2):417–30. doi: 10.1016/j.xphs.2015.11.002 26869409

[43] Moussa EM Kotarek J Blum JS Marszal E Topp EM . Physical characterization and innate immunogenicity of aggregated intravenous immunoglobulin (IGIV) in an *In vitro* cell-based model. Pharm Res (2016) 33(7):1736–51. doi: 10.1007/s11095-016-1914-4 27037576

[44] Kinderman F Yerby B Jawa V Joubert MK Joh NH Malella J . Impact of precipitation of antibody therapeutics after subcutaneous injection on pharmacokinetics and immunogenicity. J Pharm Sci (2019) 108(6):1953–63. doi: 10.1016/j.xphs.2019.01.015 30684540

[45] Kasturi SP Skountzou I Albrecht RA Koutsonanos D Hua T Nakaya HI . Programming the magnitude and persistence of antibody responses with innate immunity. Nature (2011) 470(7335):543–7. doi: 10.1038/nature09737 PMC3057367 21350488

[46] Pérez-Pérez L García-Gavín J Piñeiro B Zulaica A . Biologic-induced urticaria due to polysorbate 80: usefulness of prick test. Br J Dermatol (2011) 164(5):1119–20. doi: 10.1111/j.1365-2133.2011.10220.x 21219296

[47] McNeill IY . Hypersensitivity reaction to mannitol. Drug Intell Clin Pharm (1985) 19(7-8):552–3. doi: 10.1177/106002808501900709 2992900

[48] Pegues MA Szczepanek K Sheikh F Thacker SG Aryal B Ghorab MK . Effect of fatty acid composition in polysorbate 80 on the stability of therapeutic protein formulations. Pharm Res (2021) 38(11):1961–75. doi: 10.1007/s11095-021-03125-6 PMC8688393 34845573

[49] Dorrington MG Fraser IDC . NF-κB signaling in macrophages: Dynamics, crosstalk, and signal integration. Front Immunol (2019) 10. doi: 10.3389/fimmu.2019.00705 PMC6465568 31024544

[50] Ackland SP Hillcoat BL . Immediate hypersensitivity to mannitol: a potential cause of apparent hypersensitivity to cisplatin. Cancer Treat Rep (1985) 69(5):562–3. 3924402

[51] Spaeth GL Spaeth EB Spaeth PG Lucier AC . Anaphylactic reaction to mannitol. Arch Ophthalmol (1967) 78(5):583–4. doi: 10.1001/archopht.1967.00980030585004 6050843

[52] Findlay SR Kagey-Sobotka A Lichtenstein LM . *In vitro* basophil histamine release induced by mannitol in a patient with a mannitol-induced anaphylactoid reaction. J Allergy Clin Immunol (1984) 73(5 Pt 1):578–83. doi: 10.1016/0091-6749(84)90514-1 6201521

[53] Singh SK Mahler HC Hartman C Stark CA . Are injection site reactions in monoclonal antibody therapies caused by polysorbate excipient degradants? J Pharm Sci (2018) 107(11):2735–41. doi: 10.1016/j.xphs.2018.07.016 30055223

[54] Limaye S Steele RH Quin J Cleland B . An allergic reaction to erythropoietin secondary to polysorbate hypersensitivity. J Allergy Clin Immunol (2002) 110(3):530. doi: 10.1067/mai.2002.126460 12209107

[55] Steele RH Limaye S Cleland B Chow J Suranyi MG . Hypersensitivity reactions to the polysorbate contained in recombinant erythropoietin and darbepoietin. Nephrol (Carlton) (2005) 10(3):317–20. doi: 10.1111/j.1440-1797.2005.00389.x 15958049

[56] Weinbuch D Cheung JK Ketelaars J Filipe V Hawe A den Engelsman J . Nanoparticulate impurities in pharmaceutical-grade sugars andtheir interference with light scattering-based analysis of ProteinFormulations. Pharm Res (2015) 32(7):2419–27. doi: 10.1007/s11095-015-1634-1 PMC4452213 25630820

[57] Reich J Lang P Grallert H Motschmann H . Masking of endotoxin in surfactant samples: Effects on limulus-based detection systems. Biologicals (2016) 44(5):417–22. doi: 10.1016/j.biologicals.2016.04.012 27464990

[58] Schromm AB Paulowski L Kaconis Y Kopp F Koistinen M Donoghue A . Cathelicidin and PMB neutralize endotoxins by multifactorial mechanisms including LPS interaction and targeting of host cell membranes. Proc Natl Acad Sci (2021) 118(27):e2101721118. doi: 10.1073/pnas.2101721118 34183393 PMC8271772

[59] Fernandez SF Fung C Helinski JD Alluri R Davidson BA Knight PR . Low pH environmental stress inhibits LPS and LTA-stimulated proinflammatory cytokine production in rat alveolar macrophages. BioMed Res Int (2013) 2013:742184. doi: 10.1155/2013/742184 24288685 PMC3830824

[60] Reich J Weyer FA Tamura H Nagaoka I Motschmann H . Low endotoxin recovery–masking of naturally occurring endotoxin. Int J Mol Sci (2019) 20(4):838. doi: 10.3390/ijms20040838 PMC6412962 30781342

[61] Schwarz H Gornicec J Neuper T Parigiani MA Wallner M Duschl A . Biological activity of masked endotoxin. Sci Rep (2017) 7:44750. doi: 10.1038/srep44750 28317862 PMC5357793

[62] Bolden JS Warburton RE Phelan R Murphy M Smith KR De Felippis MR . Endotoxin recovery using limulus amebocyte lysate (LAL) assay. Biologicals (2016) 44(5):434–40. doi: 10.1016/j.biologicals.2016.04.009 27470947

[63] Bolden JS Claerbout ME Miner MK Murphy MA Smith KR Warburton RE . Evidence against a bacterial endotoxin masking effect in biologic drug products by limulus amebocyte lysate detection. PDA J Pharm Sci Technol (2014) 68(5):472–7. doi: 10.5731/pdajpst.2014.00999 25336418

[64] Galanos C Luderitz O . Electrodialysis of lipopolysaccharides and their conversion to uniform salt forms. Eur J Biochem (1975) 54(2):603–10. doi: 10.1111/j.1432-1033.1975.tb04172.x 1100380

[65] Duner KI . The importance of the quality of water in limulus amebocyte lysate tests. PDA J Pharm Sci Technol (1995) 49(3):119–21. 7613989

[66] Harm S Schildböck C Strobl K Hartmann J . An *in vitro* study on factors affecting endotoxin neutralization in human plasma using the limulus amebocyte lysate test. Sci Rep (2021) 11(1):4192. doi: 10.1038/s41598-021-83487-4 33603020 PMC7893160

[67] FDA . Guidance for industry: Immunogenicity testing of therapeutic protein products —developing and validating assays for anti-drug antibody detection. Pharm Quality/CMC (2019).

[68] FDA . Guidance for industry: ANDAs for certain highly purified synthetic peptides drug products that reference peptide drug products of rDNA origin. In: Adminsitration; FaD. U.S. Department of Health and Human ServicesSilver Spring, Maryland: Food and Drug Administration. (2021).

