# Supplementary figures and images for: Detection of innate immune response modulating impurities (IIRMI) in therapeutic peptides and proteins: Impact of excipients
Source: Front Immunol. 2022 Sep 6;13:970499. doi: 10.3389/fimmu.2022.970499 (PMC9485840; doi:10.3389/fimmu.2022.970499)

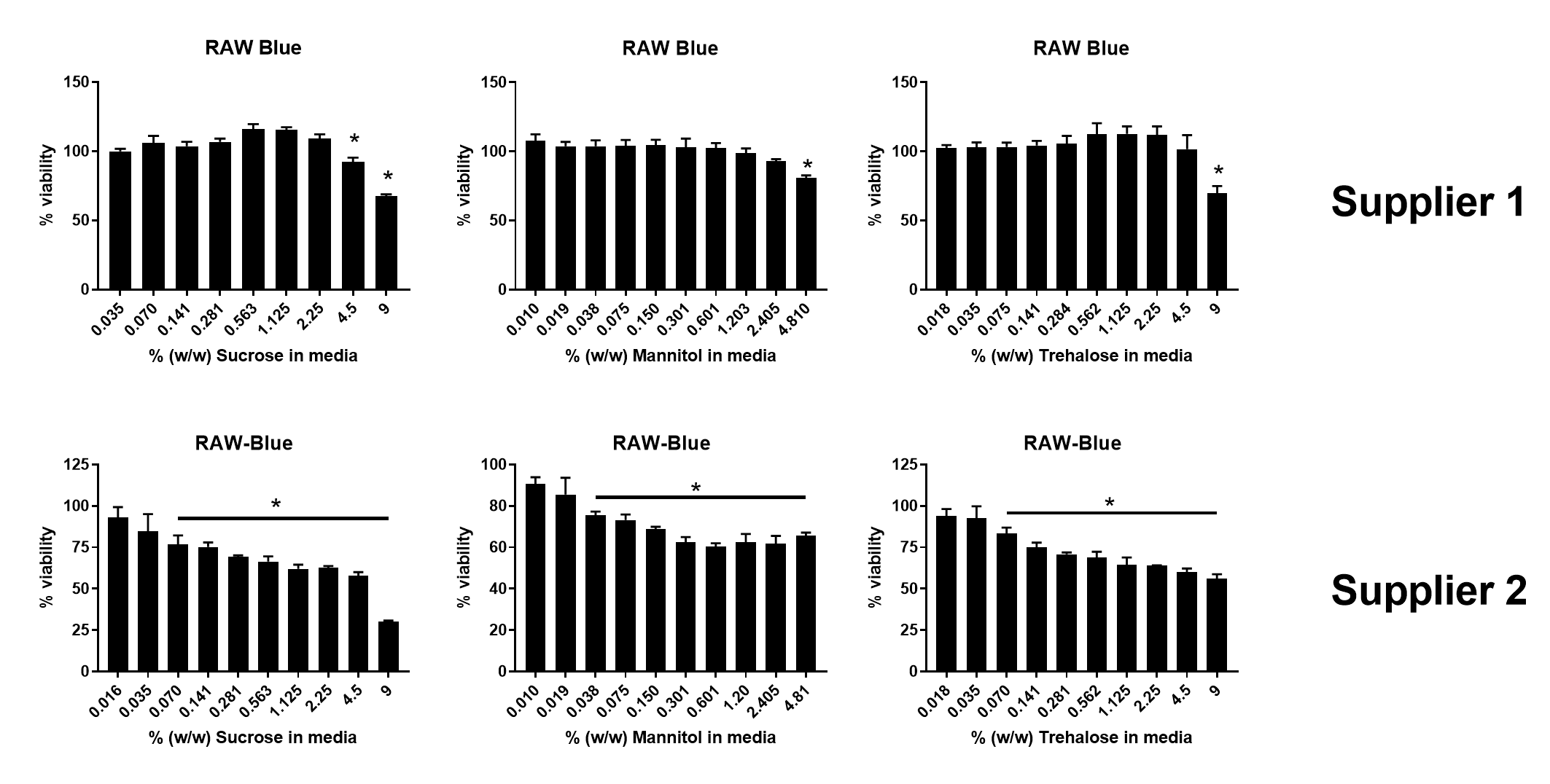

Supplement: Supplementary Figure 1 — Excipient source has an impact on cell viability. RAW-Blue cells were incubated with increasing concentrations of excipients from 2 different suppliers for 24 hours. Cell metabolism/viability was assessed using the CCK8 assay. Results are shown relative to media alone treated cells. Results are presented as the mean ± SD. * p <0.05 and ** p < 0.01. [file Image_1.tif]

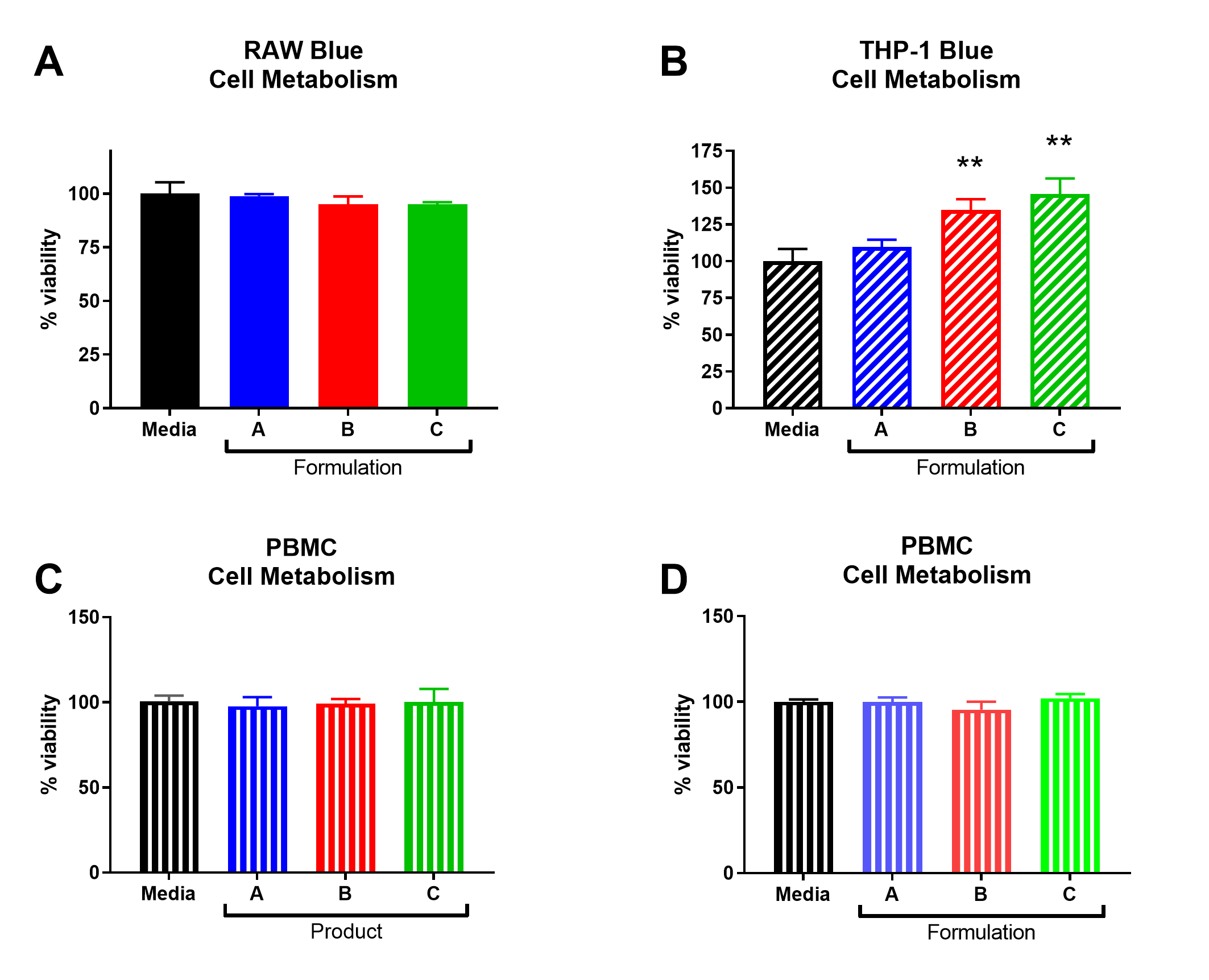

Supplement: Supplementary Figure 2 — Drug product and Drug product formulations effects on cell viability. RAW-Blue (A), THP-1-Blue (B), and PBMCs (C, D) were incubated in triplicate with the addition of 25% volume replacement of drug product or formulation alone for 24 hours. Cell lines were treated with 25% formulation volume replacement of media and PBMCs were incubated with 10% product or formulation volume replacement of media. Cell metabolism/viability was assessed using the CCK8 assay. Results are shown relative to the same cells cultured in media alone. Results are presented as the mean ± SD. RAW-, THP-1-Blue, and PBMC are solid, diagonal lines, or with vertical lines, respectively. ** p < 0.01. [file Image_2.tif]

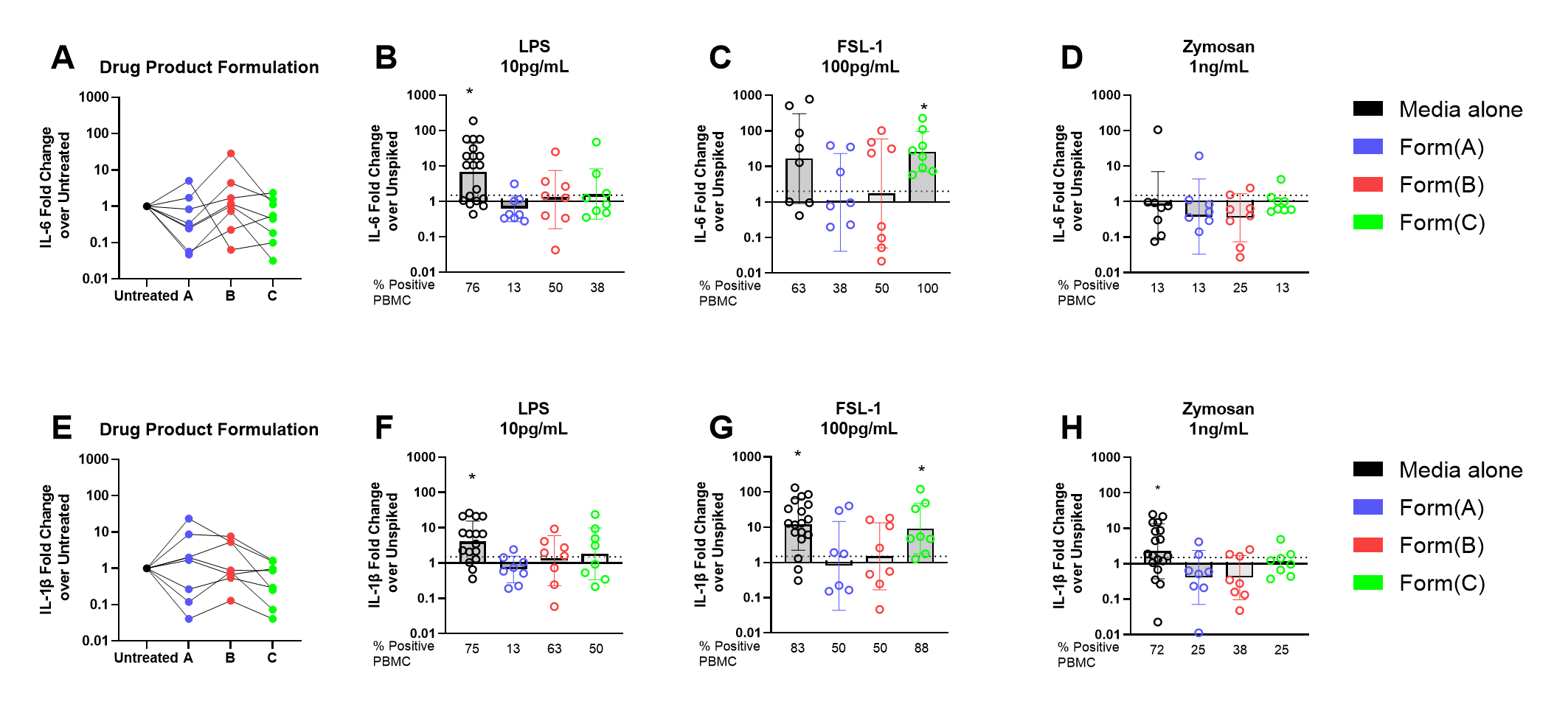

Supplement: Supplementary Figure 3 — Drug formulation components can affect PMBCs ability to respond to IIMRIs PBMCs were stimulated for 24h with LPS (10pg), FSL (100pg), or Zymosan (1ng) in media alone or the corresponding formulation (10% v/v) for 3 licensed or approved products. Fold changes in IL-6 or IL-1β gene expression are relative to the unspiked cells. (A–E) show changes in IL-6 and IL-1β expression in PBMC in the presence or absence of drug product or drug formulation (10% v/v; unspiked with TLR agonists), respectively. (B–D) show changes in IL-6 mRNA expression and F-H show changes in IL-1β in PBMC (spiked) where 10% of media was replaced by drug formulation. N = 8-18 PBMC from healthy donors per condition. Donors were considered positive if the level of IL-8 was ≥ 2-fold increase over the unspiked control; the percentage of positive PBMC in each condition is presented under each graph. * indicated p < 0.05 and is a comparison between the stimulation of cells with a model IIRMI in media or drug formulation to the corresponding unspiked control. [file Image_3.tif]
